# A lightweight UAV target detection algorithm based on improved YOLOv8s model

**DOI:** 10.1038/s41598-025-00341-7

**Published:** 2025-05-02

**Authors:** Fubao Ma, Ran Zhang, Bowen Zhu, Xirui Yang

**Affiliations:** Communication and Network Laboratory, Dalian University, Dalian, 116622 China

**Keywords:** UAV target detection, CSP-CTFN, PSC-Head, SIoU, Computer science, Information technology

## Abstract

Model lightweighting and efficiency are essential in UAV target recognition. Given the limited computational resources of UAVs and the system’s high stability demands, existing complex models often do not meet practical application requirements. To tackle these challenges, this paper proposes LW-YOLOv8, a lightweight object detection algorithm based on the YOLOv8s model for UAV deployment. First, Cross Stage Partial Convolutional Neural Network (CNN) Transformer Fusion Net (CSP-CTFN) is proposed. It integrates convolutional neural networks and a multi-head self-attention (MHSA) mechanism, and achieves comprehensive global feature extraction through an expanded receptive field. Second, Parameter Shared Convolution Head (PSC-Head) is designed to enhance detection efficiency and further minimize model size. Furthermore, the original loss function is replaced with SIoU to enhance detection accuracy. Extensive experiments on the VisDrone2019 dataset show that the proposed model reduces parameters by 37.9$\%$, computational cost by 22.8$\%$, and model size by 36.9$\%$, while improving AP, AP50, and AP75 by 0.2$\%$, 0.2$\%$, and 0.4$\%$, respectively. The results indicate that the proposed model performs effectively in UAV recognition applications.

## Introduction

Unmanned Aerial Vehicles (UAVs) are becoming increasingly important in modern technology, with broad applications in military, agriculture, environmental monitoring, disaster assessment and logistics. With advancements in UAV technology, target recognition has emerged as a crucial component in these applications^1–5^. Efficient target recognition algorithms can greatly improve UAV autonomy and task execution efficiency. However, due to the restricted endurance and limited computational capacity of UAVs, target recognition models must maintain high accuracy while being lightweight, to reduce computational burden, extend flight time, and ensure mission success^6^. Thus, this research aims to design a lightweight object detection model suitable for UAV deployment.

Current UAV target recognition methods primarily depend on deep learning-based approaches^7–9^. Deep learning-based approaches demonstrate improved performance and greater flexibility. These methods include single-stage object detection algorithms (e.g., the You Only Look Once (YOLO) series^10–15^, Single Shot Multibox Detector^16^ (SSD) ), two-stage detection algorithms (e.g., the Regions with Convolutional Neural Network (R-CNN) series^17–19^), and algorithms using attention mechanisms (e.g., Detection Transformer^20^ (DETR) ). Recently, advanced lightweight architectures such as the Multi-Scale Feature Fusion Network^21^ (LMSFF) and Lightweight Visual Mamba^22^ (LightViM) have shown significant potential for resource-constrained environments, utilizing efficient feature fusion and state-space models respectively. By learning image features automatically, these approaches greatly enhance detection accuracy and efficiency. Recent studies have further refined UAV detection techniques to address challenges in small target recognition, scale variation, and real-time performance. A small target detection model^23^ (UAV-STD) was proposed for air-to-air UAV detection, integrating an attention-based module and a spatial-scale aware prediction head to enhance detection accuracy in complex scenes. For 3D positioning, an enhanced Deformable DETR^24^ model combined with multi-view geometry improved detection robustness in occluded and scale-varying environments. Furthermore, a Spectrum-Adaptive Transformer^25^ (SAT) was developed for UAV target tracking, leveraging spatial awareness and adaptive attention to improve tracking success rates in dynamic conditions. While these advancements significantly enhance UAV detection and tracking, challenges remain in balancing detection precision, real-time performance, and computational efficiency in diverse environments.

The YOLO series has exhibited remarkable effectiveness in real-time object detection, with continuous advancements being made^26^. Specifically, the YOLOv8 model is widely favored for UAV target recognition tasks because of its high precision and rapid detection speed. However, despite its strong accuracy and speed, the intricacy of the model and its computational requirements pose difficulties for deployment in environments with limited resources, such as embedded systems or mobile devices^27^. Therefore, exploring new model architectures and optimization techniques is necessary to achieve a more optimal balance.

Therefore, this paper proposes a lightweight object detection algorithm called LW-YOLOv8 based on the YOLOv8s model for UAV deployment. LW-YOLOv8 introduces a novel Cross Stage Partial (CSP) network architecture, Cross Stage Partial Convolutional Neural Network (CNN) Transformer Fusion Net (CSP-CTFN), which integrates convolutional neural networks and multi-head self-attention mechanisms^28^ (MHSA). The input feature maps are divided into two parts based on the gradient ratio of the channels and are processed separately by the CNN and attention mechanism, optimizing computational efficiency and enhancing feature extraction. Additionally, YOLOv8 detection head performs separate computations for different object scales, leading to significant parameter and computational redundancy, which substantially increases model complexity. To address this, LW-YOLOv8 proposes the Parameter Shared Convolution Head (PSC-Head) to enhance computational efficiency by eliminating redundant operations. Furthermore, the model’s loss function is replaced with SIoU^29^ to further preserve detection accuracy and improve localization precision. These improvements collectively reduce computational load and parameter count significantly, while boosting the original model’s performance.

The main contributions of this paper are summarized as follows:

- This paper proposes a novel CSP network structure (Cross Stage Partial CNN Transformer Fusion Net) that integrates convolutional neural networks and multi-head self-attention mechanisms to optimize computational efficiency and enhance feature extraction.
- The Parameter Shared Convolution Head (PSC-Head) is introduced to reduce computational and parameter redundancy, thus lowering model complexity.
- The SIoU loss function is adopted to further refine the model’s localization accuracy and detection precision.

The rest of this paper is organized as follows: the “Methods” section details the proposed CSP-CTFN, PSC-Head, and SIoU loss function. The “Results” section provides a comprehensive evaluation of the improved model, along with comparisons to alternative models and key components. Finally, the “Conclusion” section summarizes our findings.

## Methods

The YOLO model excels in handling objects of varying scales while maintaining high detection speed, which is essential for UAV applications^30^. YOLOv8, one of the leading algorithms in the YOLO series, benefits from the careful design of each module, leveraging the strengths of classical deep learning architectures and object detection algorithms. The backbone network is based on classical CNN architectures, such as ResNet^31^ and Darknet^10^, which extract multi-scale feature maps from input images, generating features at different levels for subsequent object detection. These networks have shown excellent performance in image classification and feature extraction, making them widely used in object detection^32,33^.The neck structure is commonly used in the YOLO series and other object detection networks (e.g., Feature Pyramid Networks^34^ (FPN), Path Aggregation Networks^35^ (PANet). Inspired by Feature Pyramid Networks and Path Aggregation Networks, middle layers enhances detection across multiple scales by merging feature maps from different levels, ranging from shallow to deep layers. The detection head of YOLOv8 follows the design principles of the YOLO series and focuses on regression and classification tasks. Its design is inspired by anchor-based and anchor-free mechanisms, commonly applied in object detection systems for predicting bounding boxes (BBox) and classification scores.

### LW-YOLOv8

**Fig. 1:**
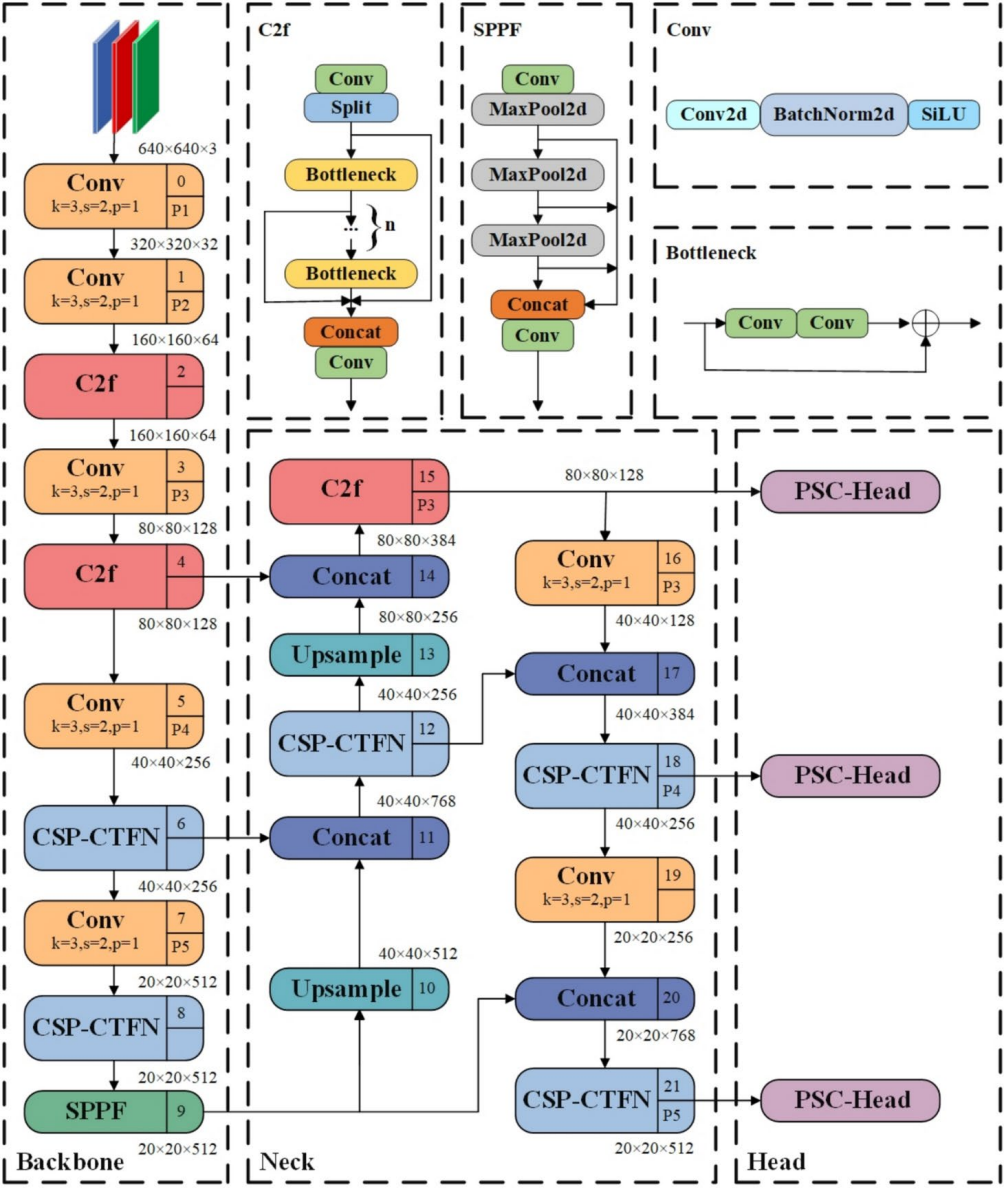
Model structure of LW-YOLOv8.

Based on CSP-CTFN, PSC-Head, and SIoU, this research enhances YOLOv8 architecture, as shown in Fig. 1. By replacing the C2f modules in layers 6, 8, 12, 18 and 21 with CSP-CTFN, substituting the detection head with PSC-Head, and incorporating the SIoU loss function, a balance between performance and computational resources can be achieved. CSP-CTFN uses fewer parameters while extracting more comprehensive image features. Replacing the detection head with PSC-Head reduces the network’s parameters while maintaining computational efficiency and ensuring accurate feature normalization, thus improving overall model efficiency. Additionally, the incorporation of the SIoU loss function, which takes into account both the shape and orientation of bounding boxes, enhances the model’s ability to detect objects with elongated shapes or non-orthogonal angles. This results in better precision for identifying irregularly shaped targets, particularly in complex UAV environments.

### CSP-CTFN

**Fig. 2:**
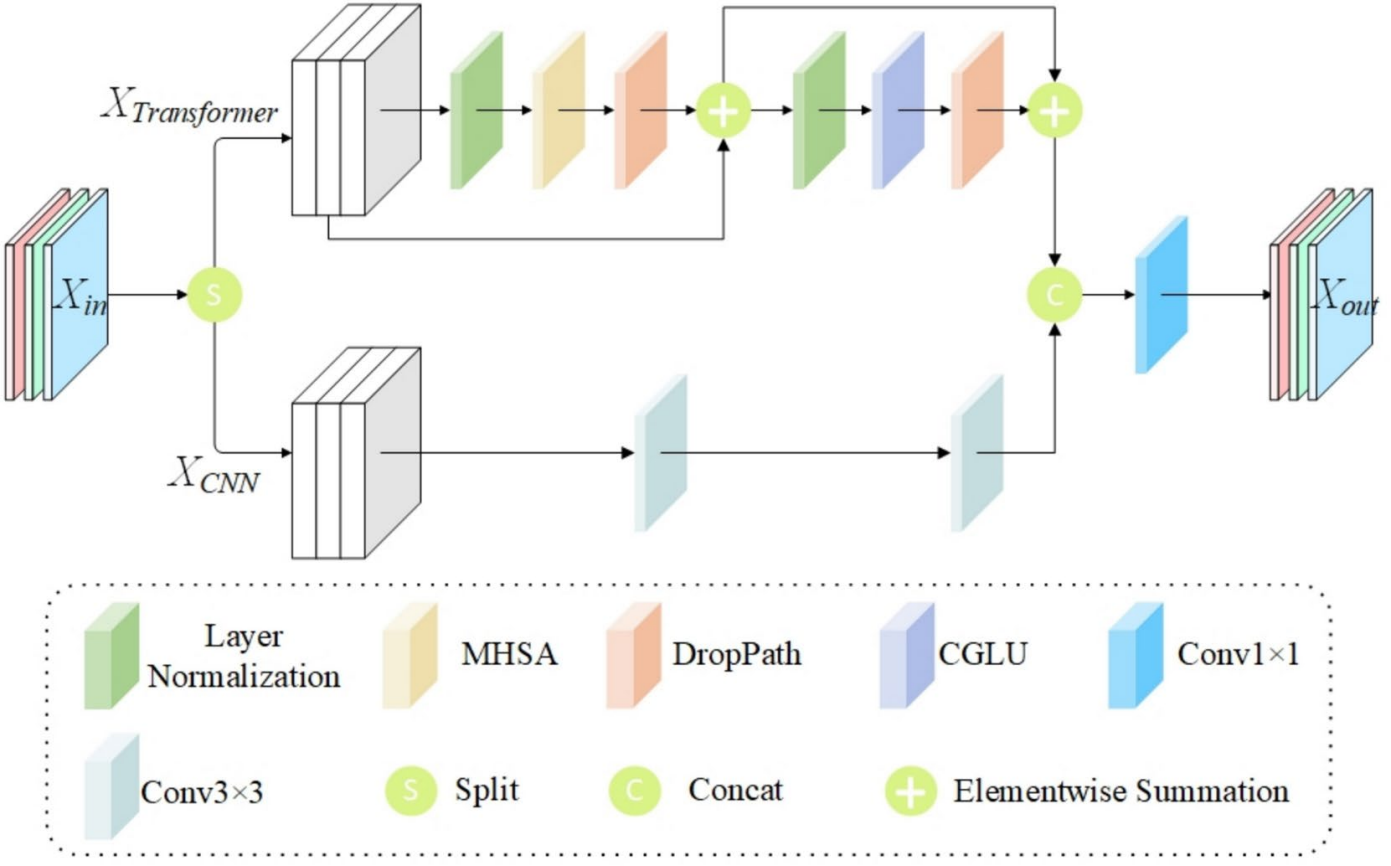
Model structure of CSP-CTFN.

Numerous studies^36–39^ have demonstrated that CNNs possess relatively small receptive fields, limiting them to capturing local features, While multi-head self-attention in Transformers is effective at capturing global features, the substantial computational complexity of the Transformer architecture results in significant overhead when applied to all channels. To ensure efficient

feature extraction while minimizing computational costs, this paper proposes a hybrid structure employing the CSP processing method. CSP divides the feature map into two sections for independent processing, then concatenates them, where one branch undergoes deep feature transformation while the other retains original features with lightweight processing. This strategic division ensures that complex transformations are applied only to part of the feature map, while the other part maintains original information through minimal processing. Additionally, the intermediate feature representation is compressed, further reducing computational cost. This dual-branch design reduces redundant computations and decreases overall computational load and memory usage. The multi-path design within the CSP structure allows gradients to flow through multiple routes, effectively reducing the risk of vanishing or exploding gradients. Additionally, the use of residual connections facilitates direct gradient propagation, which is crucial in preventing gradient vanishing and explosion, thereby enhancing training stability and boosting the model’s learning ability. The CSP-CTFN network structure is illustrated in Fig. 2.

**Fig. 3:**
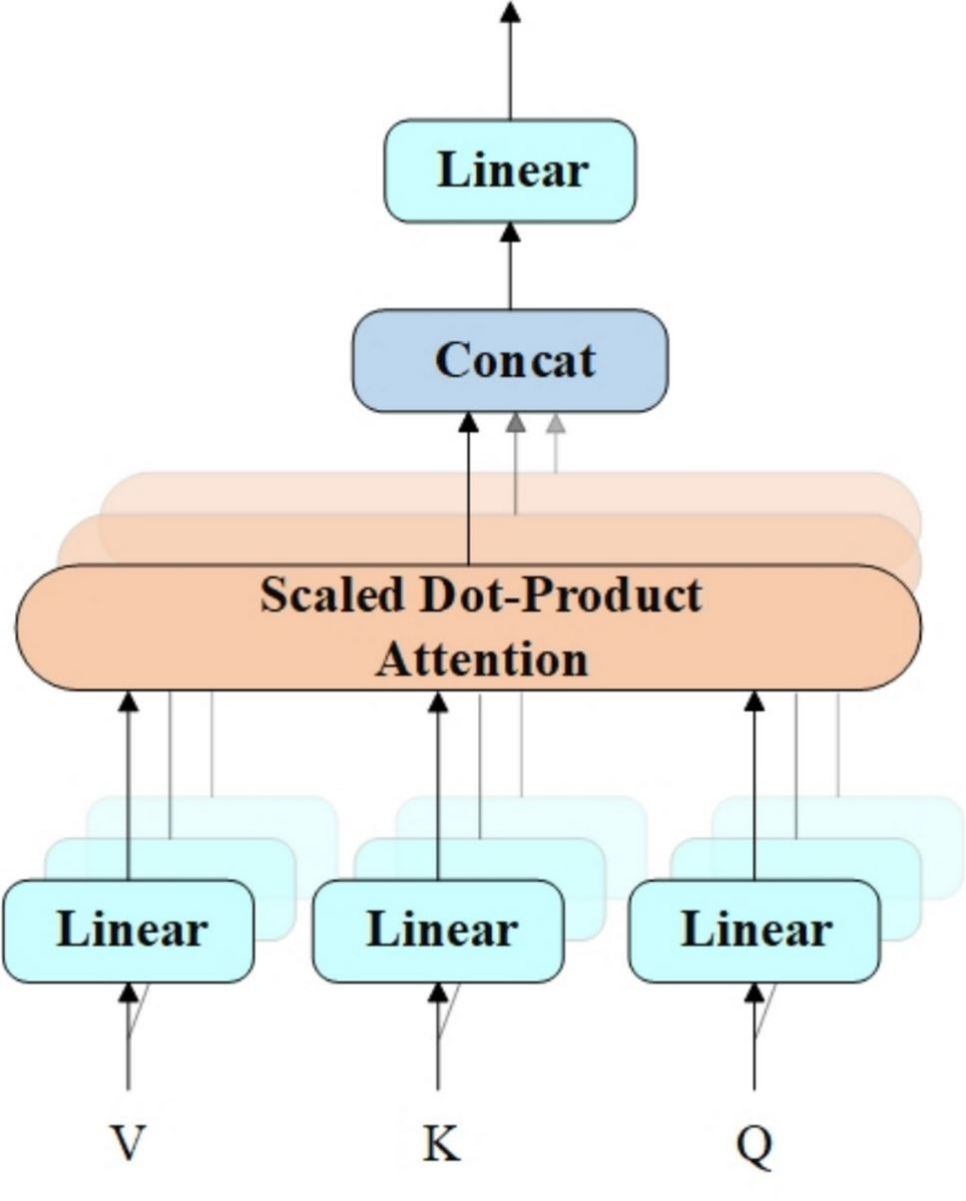
Model structure of MHSA.

**Fig. 4:**
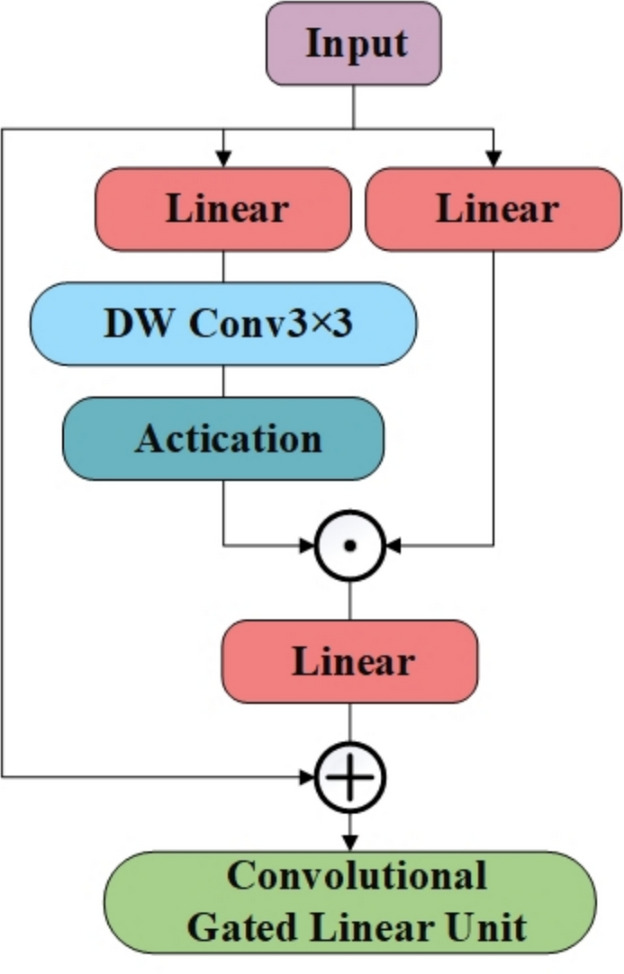
Model structure of CGLU.

Within the CSP-CTFN component, this paper integrates the Multi-Head Self-Attention mechanism with Convolutional Gated Linear Units^40^ (CGLU). MHSA extracts global features, while CGLU enhances nonlinear feature representation capabilities. CGLU offers superior performance compared to the traditional Feed-Forward Network (FFN). The parallel architecture adopted in CSP-CTFN allows for efficient GPU utilization and balanced feature extraction, ensuring a comprehensive feature representation. The structure of Multi-Head Self-Attention is illustrated in Fig. 3, while Convolutional Gated Linear Units are shown in Fig. 4.

Define the input feature map as $x\in R^{C\times H\times W}$, where C represents the number of channels, H and W denote the height and width, respectively. Initially, the feature map is divided into two components: the CNN branch and the Transformer branch.

The divided feature maps are represented as follows:

1 $$\begin{aligned} x_{CNN}&\in R^{C_{CNN}\times H\times W}\end{aligned}$$

2 $$\begin{aligned} x_{Transformer}&\in R^{C_{Transformer}\times H\times W} \end{aligned}$$

where

3 $$\begin{aligned} C_{CNN}=(1-TCR)\times C \end{aligned}$$

4 $$\begin{aligned} C_{Transformer}=TCR\times C \end{aligned}$$

*TCR* represents the channel allocation rates. To integrate the Transformer architecture for global feature extraction while minimizing the rise in computational complexity, the channel allocation to the Transformer branch is adjusted based on the number of channels and feature map size. This configuration is adapted according to the model’s depth and computational constraints, ensuring a balanced trade-off between efficiency and performance. The *TCR* value is defined as:

5 $$\begin{aligned} \begin{aligned} TCR={\left\{ \begin{array}{ll}0,C<256\\ 0.25,C=256\\ 0.5,C>256& \end{array}\right. } \end{aligned} \end{aligned}$$

For shallow layers (C < 256) with large feature maps (80$\times$80), MHSA is not applied due to prohibitive computational costs. In middle layers (C = 256) with medium feature maps (40$\times$40), *TCR* is set to 0.25 to maintain manageable computational complexity. For deep layers (C > 256) with small feature maps (20$\times$20), *TCR* increases to 0.5 to enhance global feature extraction while keeping computational costs reasonable. Next, the CNN branch processes the divided feature map using residual bottleneck blocks, as represented by:

6 $$\begin{aligned} \begin{aligned} y_{CNN}=Bottleneck(x_{CNN}) \end{aligned} \end{aligned}$$

where the Bottleneck block is employed for local feature extraction, with the output represented as $y_{CNN}$. Within the Transformer branch, input feature representations are processed using MHSA and CGLU activation functions:

7 $$\begin{aligned} y_{Transformer}&= CGLU(LayerNorm(MHSA(LayerNorm(x_{Transformer})))) \oplus x_{Transformer} \nonumber \\&\quad \oplus MHSA(LayerNorm(x_{Transformer})) \end{aligned}$$

where *MHSA* extracts global features, while *CGLU* enhances nonlinear feature representation. Finally, feature fusion is achieved by concatenating and along the channel dimension and then combining them through a convolutional layer:

8 $$\begin{aligned} \begin{aligned} y=Conv([y_{CNN},y_{Transformer}]) \end{aligned} \end{aligned}$$

where *Conv* represents the convolution operation, and the final output is denoted as $y\in R^{C\times H\times W}$.

### PSC-Head

**Fig. 5:**
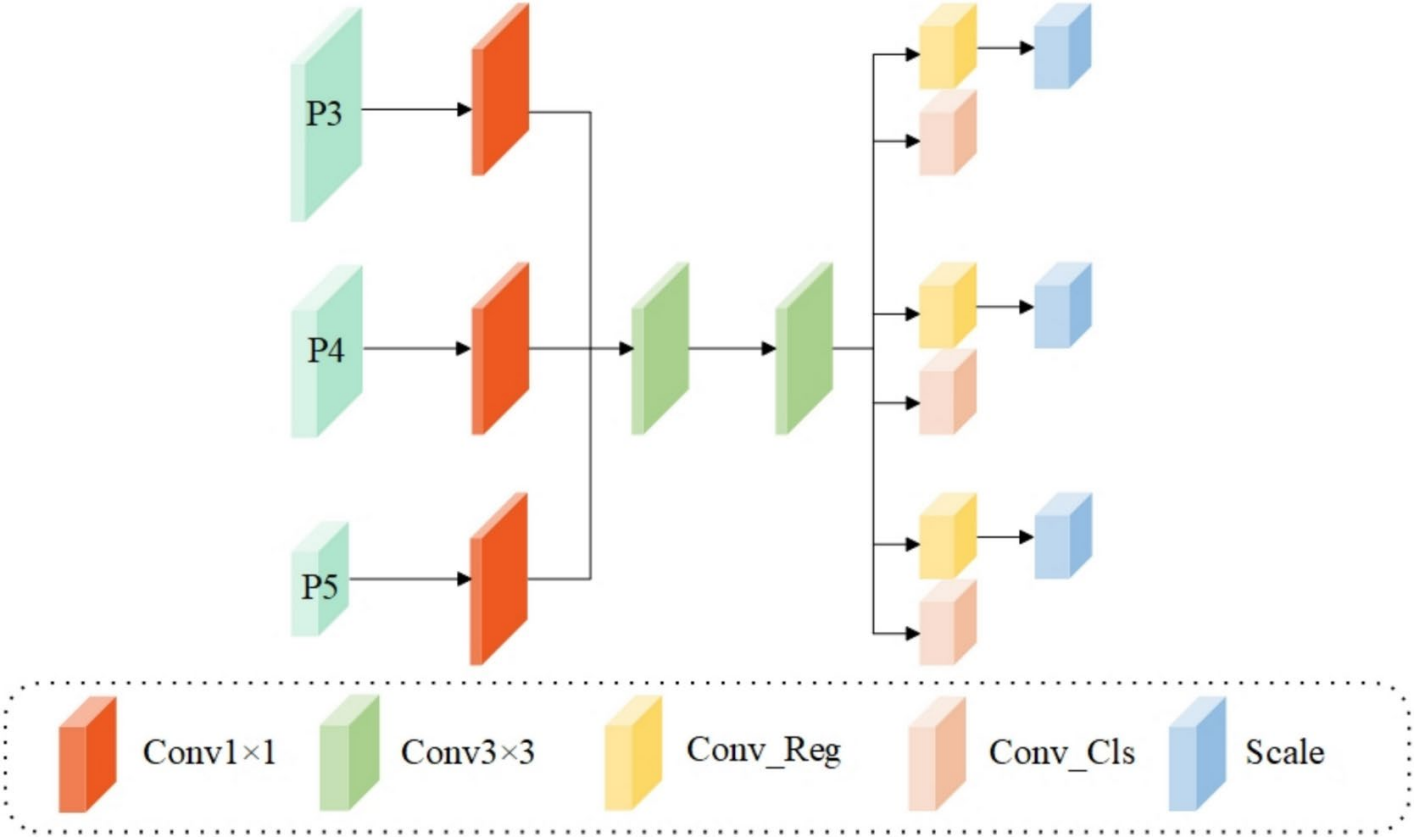
Model structure of PSC-Head.

The structure of the PSC-Head network is shown in Fig. 5. P3, P4, and P5 represent different feature pyramid levels (layers 15, 18, and 21) that process features at different scales: P3 operates on 80$\times$80 resolution features for detecting small objects, P4 processes 40$\times$40 resolution features for medium-scale objects, and P5 handles 20$\times$20 resolution features for large objects. Significant statistical differences exist across different levels of feature maps. Directly sharing parameters may lead to inconsistent feature distributions, which can negatively impact detection performance. The core idea of the PSC-Head is to minimize both the number of parameters and the computational complexity by sharing convolutional layers (green sections in Fig.5) and performing batch normalization (BN) independently (red sections in Fig. 5) to avoid sliding average value errors. This design maintains computational efficiency while ensuring accurate feature normalization.

The PSC-Head comprises three main components: a convolutional layer, a shared convolutional layer, and independent batch normalization layers. First, for each input feature map, the channel number is adjusted using a convolutional layer, as defined in Eq. (10):

9 $$\begin{aligned} \begin{aligned} X'_i=Con\nu (X_i,Chidc,1) \end{aligned} \end{aligned}$$

where Chidc signifies the total count of channels produced by the convolutional layer. Next, feature extraction is performed using a shared convolutional layer that includes two convolution operations, as shown in Eqs. (10) and (11) :

10 $$\begin{aligned} Y_i= & Con\nu 3\times 3(X_i^{\prime })\end{aligned}$$

11 $$\begin{aligned} Z_i= & Con\nu 3\times 3(Y_i) \end{aligned}$$

After each convolution, batch normalization is computed independently, followed by the application of the SiLU activation function, as detailed in Eq. (12):

12 $$\begin{aligned} \begin{aligned} Z_i=SiLU(BN(Z_i)) \end{aligned} \end{aligned}$$

Finally, the output is generated by a regression head and a classification head, which are responsible for producing regression and classification predictions, respectively, as described in Eqs. (13) and (14):

13 $$\begin{aligned} \begin{aligned} R_i=Con\nu _2(Z_i) \end{aligned} \end{aligned}$$

14 $$\begin{aligned} \begin{aligned} C_i=Con\nu _3(Z_i) \end{aligned} \end{aligned}$$

During inference, the output features from each layer are concatenated into a single tensor, followed by boundary box decoding is performed:

15 $$\begin{aligned} Box= & DecodeBboxes(R)\end{aligned}$$

16 $$\begin{aligned} Prediction= & Sigmoid(C) \end{aligned}$$

The decoded bounding boxes are then combined with the classification predictions to generate the final detection output, as described in Eq.(17):

17 $$\begin{aligned} \begin{aligned} Y=Concat(Box,Prediction) \end{aligned} \end{aligned}$$

### SIoU

Traditional IoU-based loss functions, such as GIoU^41^, DIoU^42^, and CIoU^42^, fail to consider the orientation of the predicted and ground truth boxes, which results in slower convergence. In contrast, SIoU incorporates shape and angle information of the bounding boxes during overlap calculation, enabling more precise regression for elongated or slanted objects. While the angle penalty term in SIoU requires careful weight balancing during training to ensure stable optimization, its comprehensive consideration of shape, distance, and angle information makes it particularly suitable for UAV detection scenarios. This makes the SIoU loss function particularly effective for detecting elongated or angled objects, resulting in improved performance in recognizing irregularly shaped targets in complex environments, such as those encountered by drones. To enhance the model’s bounding box regression, SIoU is applied by updating the loss computation module and integrating the SIoU formula. During training, the SIoU loss function optimized the bounding box predictions, resulting in more accurate detection results.

**Fig. 6:**
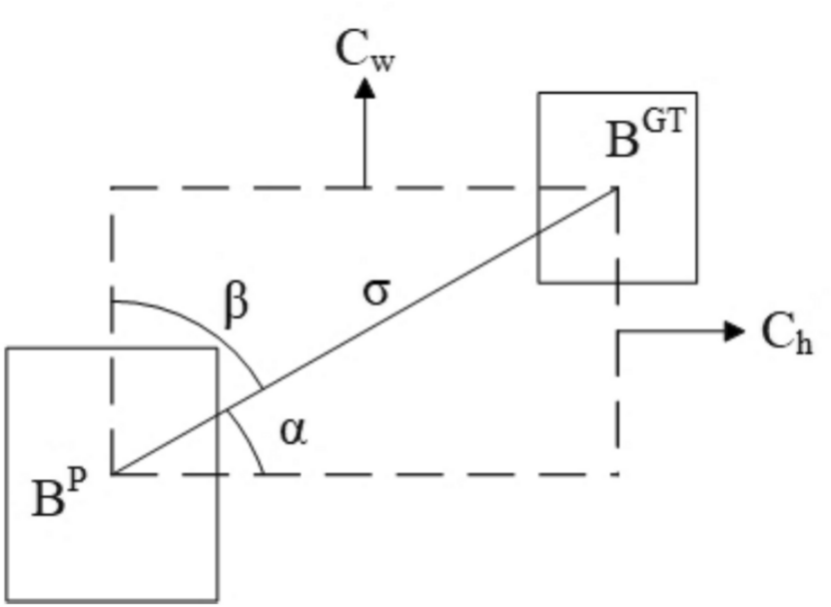
Illustration of SIoU angle loss.

The approach for computing angle loss is depicted in Fig.6, where $B^{\textrm{P}}$ stands for the predicted box, $B^{\textrm{GT}}$ indicates the ground truth box, and $C_w$, $C_h$ signify the horizontal and vertical distances between the center points of the predicted and actual boxes, respectively. When $\alpha \le \frac{\pi }{4}$, the predicted box shifts in the direction that reduces $\mathbf {\alpha }$. However, if $\alpha >\frac{\pi }{4}$, the predicted box adjusts to minimize $\varvec{\beta }$. The angle loss $\varvec{\Lambda }$ is defined as follows:

18 $$\begin{aligned} \begin{aligned} \Lambda =1-2*\sin ^2(\arcsin (x)-\frac{\pi }{4}) \end{aligned} \end{aligned}$$

where

19 $$\begin{aligned} x= & \frac{c_h}{\sigma }=\sin (\alpha )\end{aligned}$$

20 $$\begin{aligned} \sigma= & \sqrt{\left( b_{cx}^{gt}-b_{cx}^{pred}\right) ^2+\left( b_{cy}^{gt}-b_{cy}^{pred}\right) ^2} \end{aligned}$$

The coordinates ($b_{cx}^{gt}$, $b_{cy}^{gt}$) represent the center point of the ground truth box, while ($b_{cx}^{pred}$, $b_{cy}^{pred}$) denote the center point of the predicted box. The distance loss accounts for both the distance and the angle, and is defined as follows:

21 $$\begin{aligned} \begin{aligned} \Delta =\sum _{t=x,y}(1-e^{-\gamma \rho _t}) \end{aligned} \end{aligned}$$

where

22 $$\begin{aligned} \begin{aligned} \rho _x=(\frac{b_{cx}^{gt}-b_{cx}^{pred}}{C_w})^2, \rho _y=(\frac{b_{cy}^{gt}-b_{cy}^{pred}}{C_h})^2, \gamma =2-\Lambda \end{aligned} \end{aligned}$$

## Datasets and evaluation parameters

The shape loss $\Omega$ is defined as follows:

23 $$\begin{aligned} \begin{aligned} \Omega =\sum _{t=w,h}\left( 1-e^{-\omega t}\right) ^\theta \end{aligned} \end{aligned}$$

where

24 $$\begin{aligned} \begin{aligned} \omega _w=\frac{\mid w^{p}-w^{gt}\mid }{\max (w^{p},w^{gt})}, \omega _h=\frac{\mid h^{p}-h^{gt}\mid }{\max (h^{p},h^{gt})} \end{aligned} \end{aligned}$$

The parameter $\varvec{\theta }$ controls the contribution of shape loss to the overall bounding box loss, indicating the emphasis the model places on shape loss. In this paper, $\varvec{\theta }$ is set to 4. The final bounding box loss $L_{box}$ is expressed as follows:

25 $$\begin{aligned} \begin{aligned} L_{box}=1-IoU+\frac{\Delta +\Omega }{2} \end{aligned} \end{aligned}$$

where

26 $$\begin{aligned} \begin{aligned} IoU=\frac{\mid B^{\textrm{P}}\bigcap B^{GT}\mid }{\mid B^{\textrm{P}}\bigcup B^{GT}\mid } \end{aligned} \end{aligned}$$

### Datasets

This study utilized the VisDrone2019 dataset for model training and testing. The VisDrone2019 dataset is a key benchmark in drone vision, featuring numerous images captured from drone perspectives that cover various complex scenarios and multiple target types. The dataset includes 6471 training images, 548 validation images, and 1610 test images. Each image includes detailed annotations, such as target categories and bounding boxes. The dataset covers a wide range of target categories, including pedestrians, vehicles, and bicycles, across various scenarios such as urban streets, rural roads, and campuses. This dataset provides a thorough benchmark to assess the performance and efficiency of drone-based detection models. All experimental results presented in this paper are obtained using the test set of the VisDrone2019 dataset.

### Evaluation parameters

This study evaluates models using several metrics: Average Precision (AP), calculated as the mean value across various Intersection over Union (IoU) thresholds; the total number of network parameters (Params); the number of Giga Floating Point Operations per second (GFLOPs); Frames Per Second (FPS), which measures the number of images processed per second; and the model’s storage size. Precision is the ratio of correctly predicted targets to the total number of detected targets, as shown in Eq. (27). In this context, TP denotes the number of true positives, while FP represents false positives. Recall (R) is the proportion of actual targets correctly detected by the model, as given in Eq. (28), with FN representing false negatives. The mean average precision (mAP) for a single class is defined as the area under the Precision-Recall curve, shown in Eq. (29).The mean average precision for multiple classes is calculated using Eq. (30), where N denotes the number of object classes. Average Precision evaluates the mean average precision across various IoU thresholds, from 0.50 to 0.95 with a 0.05 step size. Specifically, AP50 denotes the AP at an IoU threshold of 0.5, while AP75 denotes the AP at an IoU threshold of 0.75. Additionally, Average Recall (AR100) measures the recall across all target sizes, with a maximum number of detections capped at 100. For FPS computation, a batch size of 16 is used. The FPS calculation formula is shown in Eq. (31), where preprocessing, inference, and postprocessing refer to the times for image preprocessing, inference, and postprocessing, respectively, all measured in milliseconds.

27 $$\begin{aligned} Precision= & \frac{TP}{FP+TP}\end{aligned}$$

28 $$\begin{aligned} Recall= & \frac{TP}{FN+TP}\end{aligned}$$

29 $$\begin{aligned} AP(i)= & \int _0^1P(R)dR\end{aligned}$$

30 $$\begin{aligned} AP= & \frac{1}{N}\sum _{i=1}^NAP(i)\end{aligned}$$

31 $$\begin{aligned} FPS= & \frac{1000}{preprocess+infrerence+postprocess} \end{aligned}$$

### Model training

**Fig. 7:**
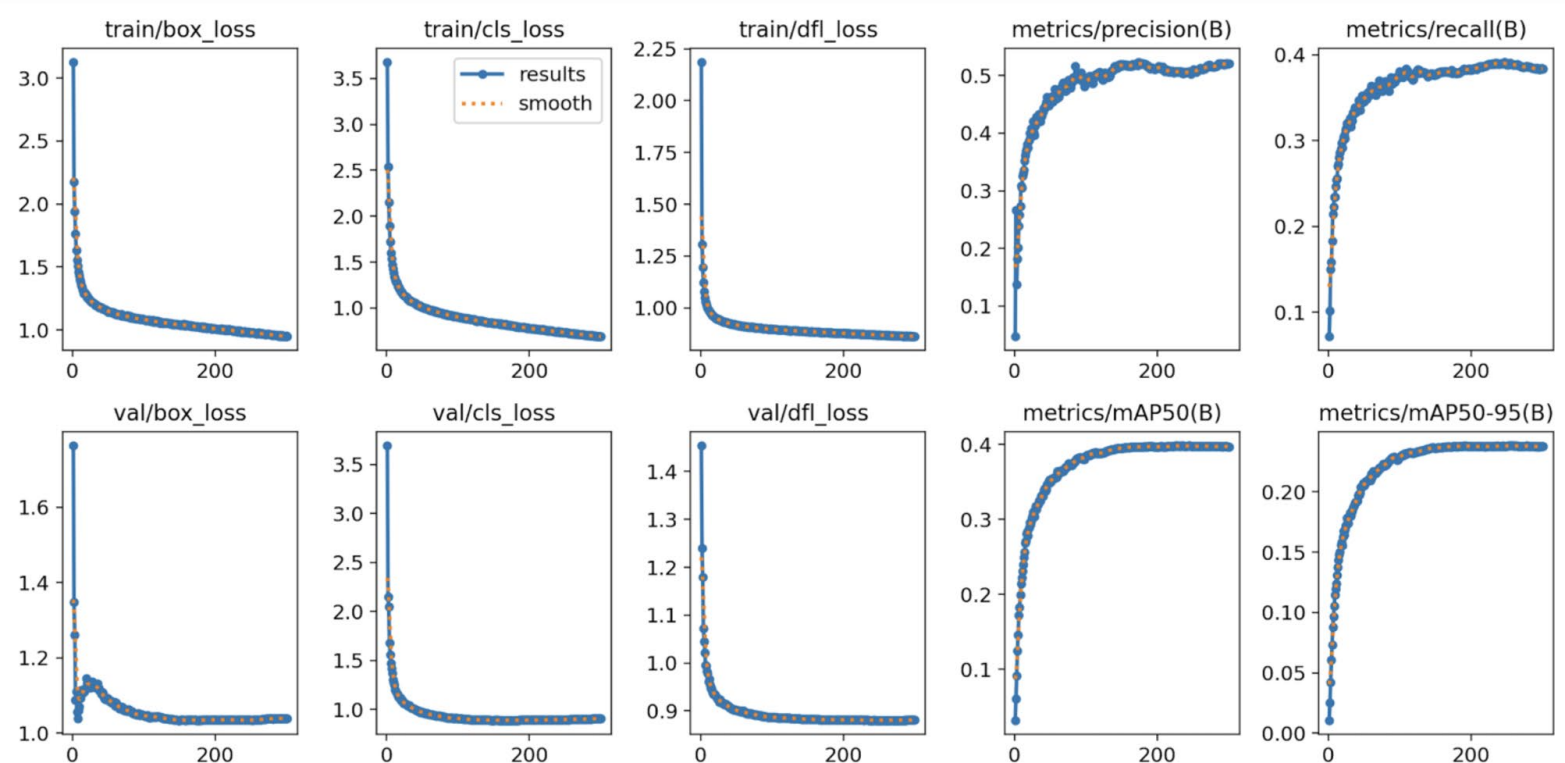
Training results of the proposed LW-YOLOv8.

In this research, model training and testing are performed on a cloud server platform equipped with an Intel(R) Xeon(R) CPU E5-2680 v4 processor, featuring 14 cores, and an RTX 3090 GPU with 24 GB of memory. The deep learning framework utilized is PyTorch 1.13.1, along with Torchvision 0.14.1. To ensure an unbiased evaluation of the model’s performance and maintain experimental fairness, no pre-trained weights are employed in any of the experiments. The training process is conducted over 300 epochs, using a batch size of 16 and an input image size of 640$\times$640 pixels. The initial learning rates (lr0 and lr1) are both set to 0.01, and the optimization is performed using SGD with a momentum of 0.937. The effectiveness of the training algorithm is further validated through experimental results, as demonstrated in Fig. 7, which illustrates the detection performance.

## Results

### Ablation experiment

To evaluate the effectiveness of the LW-YOLOv8’s improved modules, ablation experiments are conducted using the VisDrone2019 test set, as detailed in Table 1.

The improved algorithm employs a more lightweight and efficient network structure to optimize the YOLOv8s architecture. This approach improves accuracy while reducing both model parameters and computational complexity. Experimental results show that the CSP-CTFN module not only enhances accuracy but also contributes to model lightweighting. The PSC-Head module significantly enhances real-time processing and reduces model complexity, while the SIoU loss function markedly improves model accuracy. Consequently, the model’s parameters, computational cost, and size are reduced by 37.9$\%$, 22.8$\%$, and 36.9$\%$, respectively. Additionally, AP, AP50, and AP75 improved by 0.2$\%$, 0.2$\%$, and 0.4$\%$, respectively. These improvements meet the research objective of balancing lightweighting with precision.

For real-time performance, excessively low computational cost may reduce detection accuracy but enhance the algorithm’s real-time capability. Experimental results show that although the FPS of the final improved algorithm decreased slightly compared to the original, it still achieved 370.4 FPS, fully meeting real-time performance standards. Balancing accuracy and inference time better aligns with the requirements of real-world applications.

**Fig. 8:**
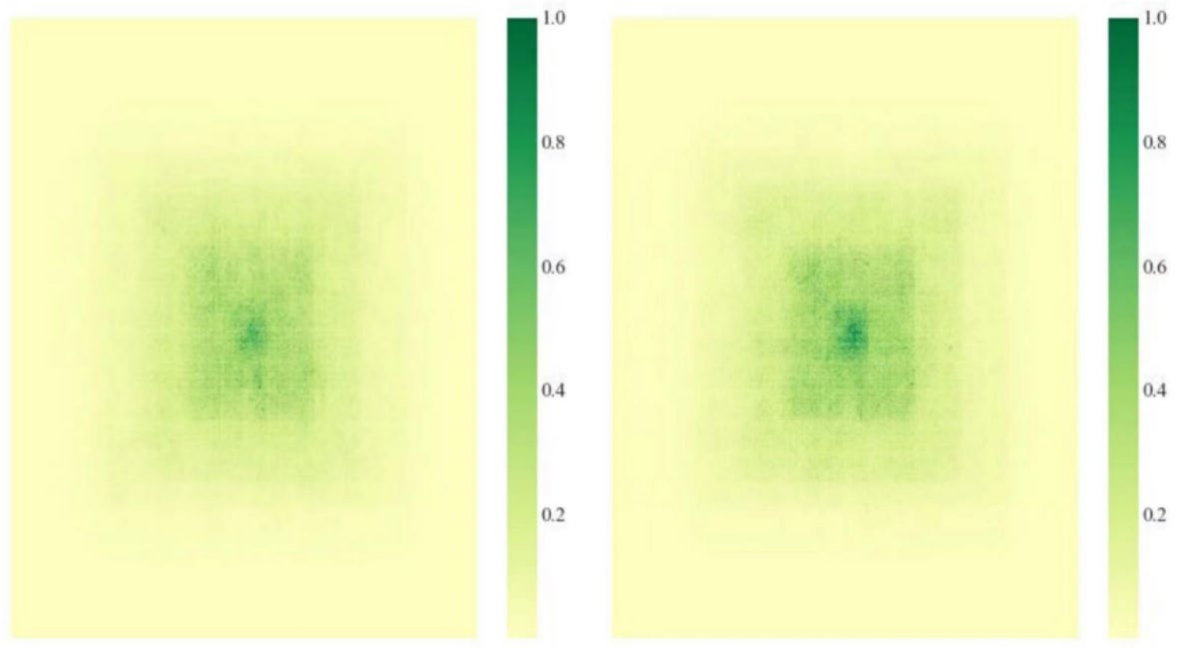
Compared the receptive fields of the backbone ( left: YOLOv8s; right: LW-YOLOv8 ).

**Fig. 9:**
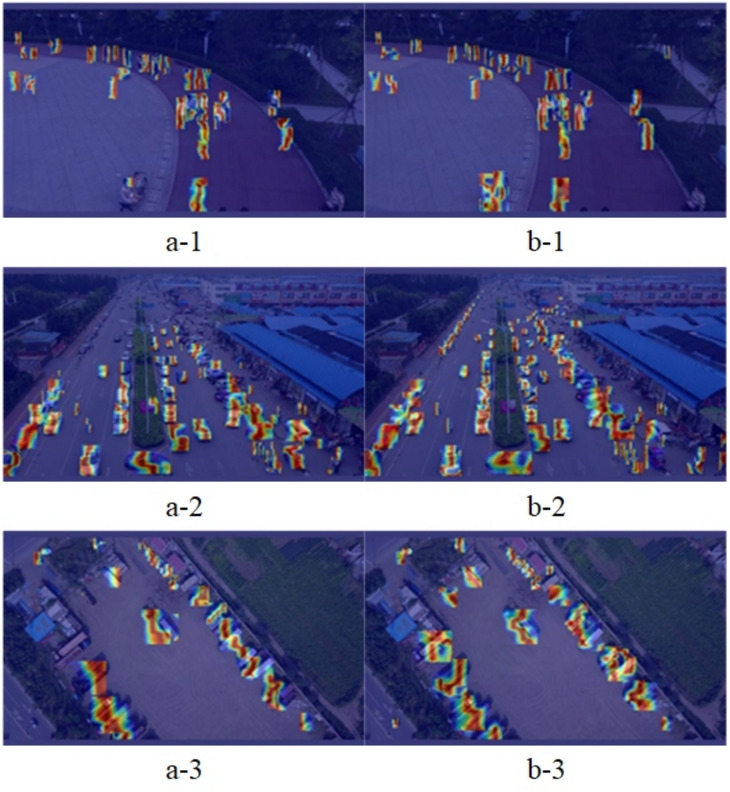
Grad-CAM visualization (a: YOLOv8s; b: LW-YOLOv8).

Figure 8 compares the receptive fields^43^ of YOLOv8s and LW-YOLOv8. At lower thresholds (e.g., 0.2, 0.3), both models exhibit similar distributions. However, as the threshold increases, LW-YOLOv8 demonstrates a larger receptive field, with a more pronounced expansion at higher thresholds (e.g., 593 vs. 543 pixels at 0.99). This indicates that LW-YOLOv8 enhances feature extraction and contextual understanding, particularly in complex environments. To offer a clearer understanding of LW-YOLOv8’s performance, Gradient-weighted Class Activation Mapping^44^ (Grad-CAM) is employed to visualize the detection results. Figure 9 compares the detection results of LW-YOLOv8 with those of the original model. Analysis reveals

**Table 1:** Ablation experiments on the different improvement components.

CSP-CTFN	PSC-Head	SIoU	AP(%)	AP$_{50}$(%)	AP$_{75}$(%)	Para (M)	FLOPs (G)	FPS	Model Size(MB)
			17.4	30.8	17.4	11.1	28.6	479.2	22.5
$\checkmark$			17.5	30.9	17.6	8.6	24.6	122.0	17.6
$\checkmark$	$\checkmark$		17.4	30.4	17.6	6.9	22.0	208.3	14.2
$\checkmark$	$\checkmark$	$\checkmark$	17.6	31.0	17.8	6.9	22.0	370.4	14.2

that LW-YOLOv8 has more precise feature focus in target areas, particularly in complex backgrounds, demonstrating greater robustness. Figure 10 compares the feature maps from stage 21 (P5 stage) between YOLOv8s (left) and LW-YOLOv8 (right). The improved model demonstrates stronger target activation (yellow highlights), better background suppression (dark purple regions), and more precise feature representation with reduced redundant activations.

### Comparison experiment on CSP-CTFN module

Table 2 presents comparative experiments between CSP-CTFN and other lightweight backbones, including RepVit^45^, StarNet^46^, FasterNet^47^, MobileNetv4^48^ and EfficientVit^49^, as well as lightweight convolutions such as GhostConv^50^, achieved by replacing the backbone and convolution layers in YOLOv8s. Experimental conditions are consistent across all experiments.

The results show that CSP-CTFN achieved the highest detection accuracy in terms of AP, AP50, and AP75, with values of 17.5$\%$, 30.9$\%$, and 17.6$\%$, respectively. Compared to EfficientViT, which has fewer parameters and lower computational complexity, CSP-CTFN improves AP by 3.4$\%$, AP50 by 4.8$\%$, and AP75 by 3.8$\%$, demonstrating a significant performance boost despite a moderate increase in FLOPs. Similarly, CSP-CTFN outperforms FasterNet and MobileNetv4, both of which have similar parameter sizes but lower detection accuracy, showing that CSP-CTFN achieves a better balance between model complexity and accuracy. Notably, StarNet has the lowest computational cost, but also the lowest AP, indicating that extreme simplification leads to a significant drop in accuracy.This improvement is attributed to its dual-branch structure, which separately captures global and local features before effectively fusing them. The global branch enhances the model’s ability to understand overall contextual information, while the local branch focuses on fine-grained details, leading to a more comprehensive feature representation and improved detection performance.

### Comparison experiment on PSC-Head

Table 3 compares the PSC-Head detection head with three other lightweight convolutional detection heads. PSC-Head achieved the highest performance in both AP and AP75, with AP50 being nearly equal to the top accuracy, differing by only 0.1$\%$. Compared to LADH-Head, PSC-Head shows notable improvements with AP increasing by 0.3$\%$ and AP75 by 0.5$\%$, particularly in high-IoU cases, demonstrating its advantage in precise localization. PSC-Head had only 0.1M more parameters

**Table 2:** Comparison experiment on CSP-CTFN.

Methods	AP(%)	AP$_{50}$(%)	AP$_{75}$(%)	Para(M)	FLOPs(G)
RepVit	16.9	30.5	16.9	11.2	29.6
Efficient-ViT	14.1	26.1	13.8	8.4	20.4
StarNet	14.1	25.6	13.8	6.5	17.3
GhostConv	16.5	29.7	16.4	10.0	26.8
FasterNet	15.7	28.2	15.6	8.6	21.7
MobileNetv4	15.6	27.8	15.7	10.6	33.9
CSP-CTFN	17.5	30.9	17.6	8.6	24.6

**Table 3:** Comparison experiment on PSC-Head.

Methods	AP(%)	AP$_{50}$(%)	AP$_{75}$(%)	Para(M)	FLOPs(G)
ScConv-Head	16.5	29.7	16.3	10.0	22.3
Efficient-Head	16.8	30.1	17.0	9.3	21.4
LADH-Head	16.8	30.1	16.8	9.3	21.5
PSC-Head	17.1	30.0	17.3	9.4	25.8

**Fig. 10:**
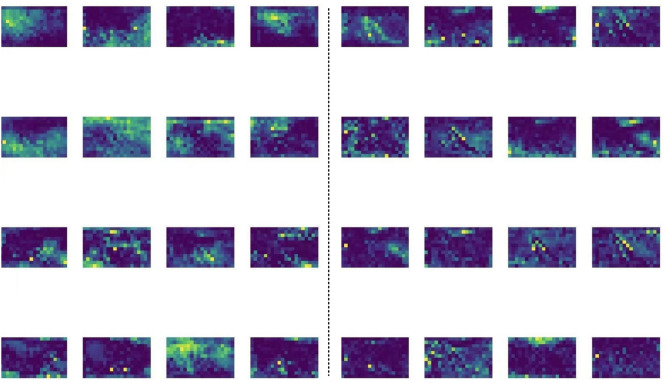
Visualization of feature maps at stage 21 (left:YOLOv8s; right: LW-YOLOv8).

than the detection head with the fewest parameters, indicating that the performance gain is primarily attributed to structural enhancements rather than excessive parameter growth. Although PSC-Head incurs slightly higher computational costs compared to LADH-Head (increasing from 21.5G to 25.8G FLOPs), the significant improvement in detection accuracy, especially for high-precision detection scenarios (AP75), makes it a more advantageous choice. The performance improvement can be attributed to the use of shared convolutional layers, which reduces the number of parameters and computational complexity. Additionally, performing batch normalization independently helps to avoid sliding average errors, leading to more precise normalization and enhanced overall detection accuracy.

**Table 4:** Comparison experiment on different loss function.

Methods	AP(%)	AP$_{50}$(%)	AP$_{75}$(%)	FLOPs(G)
CIoU	17.4	30.4	17.6	32.5
EIoU^51^	17.3	30.7	17.4	32.4
GIoU	17.4	30.7	17.7	32.6
DIoU	17.4	30.9	17.6	32.4
WIoUv3^52^	17.5	30.9	17.8	32.5
MPDIoU^53^	17.4	30.8	17.6	32.3
SIoU	17.6	31.0	17.8	32.6

**Table 5:** Comparison experiment on different models.

Methods	AP(%)	AP$_{50}$(%)	AP$_{75}$(%)	Para(M)	FLOPs(G)
YOLOv5s	13.1	26.0	12.0	7.0	15.8
YOLOv6s	17.1	29.7	17.6	18.5	45.2
YOLOv7-Tiny	15.4	29.8	14.3	6.0	13.2
YOLOv8s	17.4	30.8	17.4	11.1	28.6
Faster-RCNN	19.7	33.2	21.0	41.4	208.0
YOLOv10s^54^	17.1	30.3	17.2	8.0	24.5
YOLOv11s^55^	16.9	29.9	17.0	9.4	21.3
LW-YOLOv8	17.6	31.0	17.8	6.9	22.0

### Comparison experiment on different loss function

Table 4 provides a detailed comparison of various loss functions. To enhance model accuracy without increasing parameters or computational load, several loss functions are tested under consistent conditions. This experiment specifically aims to assess the suitability of SIoU by comparing it with six other bounding box regression loss functions, including CIoU used in YOLOv8, as shown in Table 4. The performance improvement can be attributed to SIoU’s explicit modeling of angle alignment, which is particularly beneficial for aerial imagery where objects often appear in oblique views and irregular orientations. The performance improvement is due to SIoU’s incorporation of shape and angle information of the bounding boxes during overlap calculation. This allows for more precise regression, particularly for elongated or slanted objects, resulting in better alignment and higher detection accuracy.

The results show that SIoU outperforms CIoU, EIoU, GIoU, DIoU, WIoUv3, and MPDIoU in AP, AP50, and AP75, with values of 17.6$\%$, 31.0$\%$, and 17.8$\%$, respectively. Additionally, SIoU achieves the highest average recall across various IoU thresholds and target sizes, with a maximum detection number of 100, reaching 32.6$\%$.

### Comparison experiment on different models

Table 5 compares the improved algorithm proposed in this paper with other algorithms. The comparison includes YOLOv5s, YOLOv6s, YOLOv7-Tiny, YOLOv8s, Faster-RCNN, YOLOv10s and YOLOv11s.

The results show that LW-YOLOv8 achieved competitive performance among YOLO series algorithms (YOLOv5s, YOLOv6s, YOLOv7-Tiny, YOLOv8s, YOLOv10s, and YOLOv11s), with AP of 17.6$\%$, AP50 of 31.0$\%$, and AP75 of 17.8$\%$, showing improvements of 0.2$\%$, 0.2$\%$, and 0.4$\%$ over YOLOv8s, and even greater enhancements over other YOLO variants. Compared to Faster-RCNN in terms of detection accuracy, the proposed algorithm is lower by 2.1$\%$, 2.2$\%$, and 3.2$\%$ in AP, AP50, and AP75 respectively. However, the parameter count and computational load of LW-YOLOv8 are significantly lower than Faster-RCNN, with 83.3$\%$ fewer parameters and 89.4$\%$ lower FLOPs. The proposed LW-YOLOv8 algorithm successfully balances accuracy and computational complexity, making it highly efficient.

## Conclusion

This paper presents an improved lightweight object detection algorithm based on YOLOv8s for UAV target recognition, addressing the limitations of traditional YOLOv8s in such applications. First, a novel CSP network structure, CSP-CTFN, combining convolutional neural networks with a multi-head self-attention mechanism, is proposed. This network enhances global feature extraction by expanding the receptive field. Second, a parameter-sharing detection head (PSC-Head) is introduced, building on existing lightweight heads to enhance detection efficiency and further lower model complexity. Lastly, SIoU is substituted for the original loss function, resulting in a significant boost in detection accuracy.

Experiments conducted on the VisDrone2019 dataset show that LW-YOLOv8 offers low computational overhead and high efficiency, fulfilling the real-time demands of UAV target recognition. The model is particularly well-suited for specific UAV application scenarios including urban surveillance, traffic monitoring, search and rescue operations, and agricultural inspection. Its strengths lie in the balanced trade-off between accuracy and computational requirements, making it viable for deployment on edge computing devices commonly used in commercial UAVs. Despite these improvements, LW-YOLOv8 still has some limitations. First, deployment on extremely resource-constrained UAVs may still require further optimization. Second, the model has been primarily tested on the VisDrone2019 dataset, which focuses on urban drone-based target detection; its performance in more complex environments, such as nighttime or adverse weather conditions, requires further investigation. Third, while purposed model performs well on the dataset tested, it may face challenges when detecting densely packed or overlapping objects, which are common in crowded UAV surveillance scenarios. Lastly, additional techniques such as neural architecture search (NAS) or knowledge distillation could be explored to enhance efficiency without sacrificing accuracy.

## Data Availability

The dataset used in this study is the publicly available VisDrone2019 dataset, which can be accessed at https://github.com/VisDrone/VisDrone-Dataset. This dataset is widely used for UAV-based object detection tasks, ensuring reproducibility and allowing for further research and validation.
